# Challenges to the Pair Bond: Neural and Hormonal Effects of Separation and Reunion in a Monogamous Primate

**DOI:** 10.3389/fnbeh.2016.00221

**Published:** 2016-11-14

**Authors:** Katie Hinde, Chelsea Muth, Nicole Maninger, Benjamin J. Ragen, Rebecca H. Larke, Michael R. Jarcho, Sally P. Mendoza, William A. Mason, Emilio Ferrer, Simon R. Cherry, Marina L. Fisher-Phelps, Karen L. Bales

**Affiliations:** ^1^California National Primate Research Center, University of CaliforniaDavis, CA, USA; ^2^School of Human Evolution and Social Change, Arizona State UniversityTempe, AZ, USA; ^3^Center for Evolution and Medicine, Arizona State UniversityTempe, AZ, USA; ^4^Department of Psychology, University of CaliforniaDavis, CA, USA; ^5^Human Development and Family Studies, Pennsylvania State UniversityPennsylvania, PA, USA; ^6^Department of Psychology, Siena CollegeLoudonville, NY, USA; ^7^Department of Biomedical Engineering, University of CaliforniaDavis, CA, USA; ^8^Department of Biological Sciences, Texas Tech UniversityLubbock, TX, USA

**Keywords:** attachment, separation, stress, cortisol, oxytocin, vasopressin

## Abstract

Social monogamy at its most basic is a group structure in which two adults form a unit and share a territory. However, many socially monogamous pairs display attachment relationships known as pair bonds, in which there is a mutual preference for the partner and distress upon separation. The neural and hormonal basis of this response to separation from the adult pair mate is under-studied. In this project, we examined this response in male titi monkeys (*Callicebus cupreus*), a socially monogamous New World primate. Males underwent a baseline scan, a short separation (48 h), a long separation (approximately 2 weeks), a reunion with the female pair mate and an encounter with a female stranger (with nine males completing all five conditions). Regional cerebral glucose metabolism was measured via positron emission tomography (PET) imaging using [18F]-fluorodeoxyglucose (FDG) co-registered with structural magnetic resonance imaging (MRI), and region of interest (ROI) analysis was carried out. In addition, plasma was collected and assayed for cortisol, oxytocin (OT), vasopressin (AVP), glucose and insulin concentrations. Cerebrospinal fluid (CSF) was collected and assayed for OT and AVP. We used generalized estimating equations (GEE) to examine significant changes from baseline. Short separations were characterized by decreases in FDG uptake, in comparison to baseline, in the lateral septum (LS), ventral pallidum (VP), paraventricular nucleus of the hypothalamus (PVN), periaqueductal gray (PAG), and cerebellum, as well as increases in CSF OT, and plasma cortisol and insulin. Long separations differed from baseline in reduced FDG uptake in the central amygdala (CeA), reduced whole brain FDG uptake, increased CSF OT and increased plasma insulin. The response on encounter with a stranger female depended on whether or not the male had previously reproduced with his pair mate, suggesting that transitions to fatherhood contribute to the neurobiology underlying response to a novel female. Reunion with the partner appeared to stimulate coordinated release of central and peripheral OT. The observed changes suggest the involvement of OT and AVP systems, as well as limbic and striatal areas, during separation and reunion from the pair mate.

## Introduction

Social bonds form the foundation of our daily lives, and are now widely acknowledged to have far-reaching effects on health and psychological well-being (Uchino, 2006; Cacioppo et al., 2015; Valtorta et al., 2016). While these social relationships may take many forms including friendships, parent-offspring relationships, and status or power relationships, as adults our closest relationship is usually with a romantic partner. Pair bonds are forms of attachment relationships (Hazan and Shaver, 1987); first studied in mothers and offspring (Bowlby, 1969; Ainsworth et al., 1978), these attachment relationships include a strong preference for the familiar partner, distress upon separation from the partner and the ability of the partner to buffer the individual against stress (Mason and Mendoza, 1998).

Titi monkeys (*Callicebus cupreus*), socially monogamous New World monkeys, form strong adult pair bonds. They show a behavioral preference for their partner over a stranger (Carp et al., 2016); their partner can buffer their stress response to novelty (Hennessy et al., 1995; Mendoza et al., 2000); and they display distress upon separation from their partner, which is not reduced by the presence of another animal (Mendoza and Mason, 1986a,b; Mendoza, 1991; Laugero et al., 2011; Ragen et al., 2012). Distress is displayed both physiologically by increased cortisol concentrations and behaviorally by contact calls for the mate and increased locomotion (Mendoza et al., 2000; Ragen et al., 2012). Separation thus presents a challenge to the pair bond; and while impaired negative feedback appears to keep cortisol concentrations high for an extended period (Mendoza et al., 2000), titi monkeys will also form new pair bonds if their original mate is gone (Van Belle et al., 2016).

The physiological consequences of separation from a pair mate include the increased cortisol concentrations described above; however, changes in other hormones and in neural substrates are less studied. Oxytocin (OT) and vasopressin (AVP) are nine-amino acid peptides synthesized in the paraventricular (PVN) and supraoptic (SON) nuclei of the hypothalamus (Zingg, 2002). They are intimately involved in pair bonding in prairie voles (*Microtus ochrogaster*), a socially monogamous rodent (Carter, 1998; Young et al., 2005; Gobrogge and Wang, 2015; Johnson et al., 2016; Numan and Young, 2016), and have been implicated in studies of adult human romantic relationships (Gordon et al., 2008; Walum et al., 2008, 2012; Scheele et al., 2012, 2013; Schneiderman et al., 2014). In human studies, OT and AVP are almost exclusively measured in blood (or other peripheral fluids). Plasma OT and AVP may or may not be reflective of brain activity, depending on context and timing of the blood sample (Landgraf and Neumann, 2004; Freeman et al., 2016a); cerebrospinal fluid (CSF) concentrations are generally understood to be more reflective of central nervous system levels (Born et al., 2002; Landgraf and Neumann, 2004).

Cortisol is a metabolic hormone, and it (as well as other metabolic hormones such as glucose and insulin) may change in response to challenging social circumstances (Mendoza, 2017). These challenging social circumstances could include negative events such as loss of close adult female kin due to predation which can lead to elevated circulating cortisol for several weeks (Engh et al., 2006). Social situations we think of as positive may also be challenging metabolically. For instance, male prairie voles lose significant amounts of weight when caring for their first litter, demonstrated by drops in fat depots and circulating leptin (Campbell et al., 2009), as well as plasma insulin (Conley, 2012). In socially monogamous California mice, male body mass decreased when paired with a breeding female, and increased during their pair mate’s pregnancies (Saltzman et al., 2015). Male tamarins and marmosets gain weight during their mates’ pregnancies (Ziegler et al., 2006; Sánchez et al., 2008) and lose weight during periods of infant care (Sánchez et al., 1999). Social dynamics that present psychological and metabolic challenges—the absence of social partners, greater social responsibility in the form of pair bond maintenance or parenting behavior, or the presence of unexpected individuals—can all influence the physiological regulation of the individual.

Involuntary separation from an attachment figure, if it continues, may cause significant distress (Sun et al., 2014). In male prairie voles, chronic separation from the pair mate resulted in increased plasma corticosterone and increased OT, AVP and corticotrophin-releasing hormone (CRH) in the PVN of the hypothalamus; however, plasma OT and AVP did not change (Bosch et al., 2009; McNeal et al., 2014; Sun et al., 2014). This differs from human studies in which challenges to the pair bond or grief at the loss of a loved one, appear to result in sex-specific changes in plasma OT and AVP (Taylor et al., 2000, 2010). Human studies on the neural correlates of grief have implicated the cingulate cortex, the thalamus and the brainstem (Gundel et al., 2003; Kersting et al., 2009), as well as reward areas such as the nucleus accumbens (NAcc; O’Connor et al., 2008).

In this study, we examined the neural and hormonal correlates of both acute and chronic separation from the pair mate in adult male titi monkeys. Our overall hypothesis was that pair bonded males would show short and long term adaptions to separation in neural and hormonal systems engaged in emotion, stress, and motivation; and that a reunion with the familiar pair mate would have different significance than an encounter with a new female. We included a number of neural areas based on human and animal studies (see “Materials and Methods” Section). We predicted that males would display increased plasma cortisol and plasma AVP in both long and short separations, as well as activation of areas associated with “social pain” such as the cingulate cortex (Eisenberger, 2015). We predicted the involvement of reward pathways, such as the NAcc and ventral pallidum (VP), both because they have been implicated in human and vole studies of grief (O’Connor et al., 2008; Bosch et al., 2016), and because they are responsive to aversive as well as appetitive circumstances (Roitman et al., 2005; Saga et al., 2016; Soares-Cunha et al., 2016). We examined areas that either produce or have receptors for OT and AVP in this species [PVN; SON of the hypothalamus; lateral septum (LS)]. We also examined the correlates of reunion with the partner vs. a female stranger, and predicted that we would see responses to the mate but not the stranger, especially in PVN, SON and LS. Finally, we also hypothesized that parenting in a monogamous primate can alter the salience of an unfamiliar female as a potential new mate. Based on evidence from prairie voles that pair bonding may be facilitated by reproduction (Resendez et al., 2016), we compared responses to reunion as a function of whether or not the pair had produced offspring together prior to the separation, predicting increased neural uptake in fathers in the PVN, SON and LS.

## Materials and Methods

### Subjects

We studied 12 adult males born and housed at the California National Primate Research Center, all of which had female pair mates. All animals were fed twice daily; details of husbandry, training and caging are identical to those described in Mendoza (1999) and Tardif et al. (2006). The mean age of subjects was 5.8 years (range 2.9–8.7), and the mean ± SD duration since pairing was 1.02 ± 1.0 years (range 0.3–3.33 years). Subjects participated in five separation and partnership conditions designed to compare how the length of separation (either long-term or short-term) affected [18F]-fluorodeoxyglucose (FDG) uptake in the central nervous system and both central and peripheral hormone concentrations. As the brain uses glucose for fuel, FDG uptake is a proxy for brain activity. Five of the 12 males were fathers for at least some of the conditions.

All procedures in this study were approved by the Animal Care and Use Committee of the University of California, Davis, and complied with National Institutes of Health ethical guidelines as set forth in the Guide for Lab Animal Care.

### Conditions

**Figure 1:**
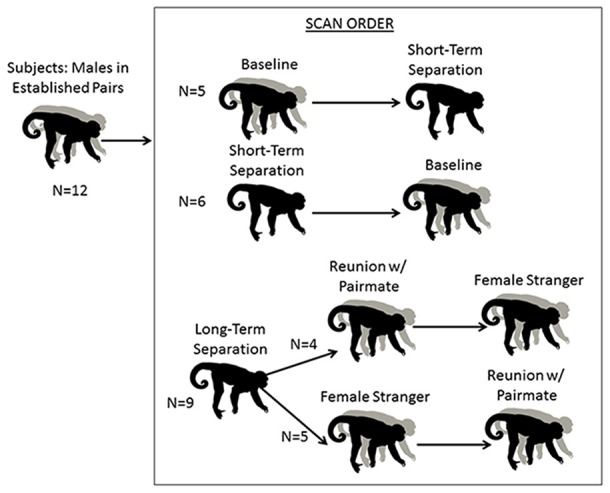
**Schematic of research design.** Males underwent five conditions. The first two were a baseline scan while with their pair mate, and a short separation of 48 h. These two conditions were counterbalanced. All males then underwent a 2-week long “long separation” from their pair mate. They then underwent two final scans, following an encounter with a stranger female or a reunion with their pair mate (these two scans were also counterbalanced).

Male subjects underwent separation and partnership conditions with concurrent blood sampling and brain scans, and pair mates were absent or present accordingly (Figure 1; Table 1). The normal housing for all males was living with their pair-mates. A “baseline” scan was therefore under their normal housing conditions; whereas a “short separation” scan was after having been separated from the mate for 48 h. These were counter-balanced in the sense that half of the males had the baseline scan first, and half of the males had the short separation scan first. If they underwent the short separation first, after that scan they were returned to their pair-mate, waited at least a week to 10 days, and then underwent the baseline scan. Separations were not isolation; all animals had visual, olfactory and vocal communication with other titi monkeys throughout the separation period.

**Table 1:** **Social conditions for subject males when in the home cage and during the 30 min conscious uptake during the positron emission tomography (PET) scan**.

	Home cage social condition	FDG uptake social condition
Baseline	With pair mate	With pair mate
Short separation	Alone	Alone (after 48 h of separation)
Long separation	Alone	Alone (after 2	#x02013;3 weeks of separation)
Partner reunion	Alone	With pair mate
Stranger	Alone	With stranger female

Following the baseline and short separation scans, subjects were then separated from their pair mates for approximately 2–3 weeks (*n* = 9, mean = 15.78 days, SD = 3.53 days, range = 14–22 days), after which “long-term separation” scans and hormone measures were taken. This was followed by two final counterbalanced conditions: reunion with the pair mate and encounter with a female stranger. This experimental protocol afforded us the ability to disentangle the specific response of reunion with the pair mate as compared to the more generic response to any female. Four of the males had the partner reunion scan first and then the stranger scan, and the other five males had the stranger scan first then the partner reunion scan. Time spent separated between the long-term separation scan, and the first of these two scans (*n* = 9) was a mean of 11.11 days (SD = 6.05, range = 7–26 days). One male’s scan had to be redone. When he is excluded, the time spent separated was a mean of 9.25 days (SD = 2.49, range = 7–14, *n* = 8). The two final scans (partner reunion and stranger scans) were a mean of 17.22 days apart (SD = 25.87, range = 6–86 days). Eight of the nine subjects had a reunion and stranger scans a mean of 8.63 days apart (SD = 2.13, range = 6–12 days). One of the subjects had his stranger scan done 86 days prior to the reunion scan with the pair mate; this male was separated from his pair mate for 21 days prior to the stranger scan and 22 days prior to his reunion scan with his pair mate.

Out of 12 subjects, data were collected on all but three subjects (*n* = 9) at all five conditions. Data on two subjects were collected only at baseline and short-term separation, and data on one subject were collected only at baseline. In addition, cases of missing hormone data varied for individuals at each measurement occasion, due to sensitivity of the hormone assays (particularly for CSF samples, in which concentrations were near the bottom of some assays) and the difficulty of obtaining CSF samples (described below).

### Outcome Measures

#### PET Scan with FDG

Positron emission tomography (PET) scans were administered to all subjects. Forty-eight hours prior to scans, subjects (together with pair mates and offspring less than 1 year old, if they were in baseline condition; alone if they were in any other condition; Table 1) were relocated to a metabolism room, to reduce possible effects of novel housing on brain metabolism. On the day of the PET scan, male subjects were caught and removed from the cage. Subjects were restrained, a blood sample was collected for another investigation (Jarcho in progress), and then subjects received a bolus [^18^F]-FDG (PETNET Solutions, Sacramento, CA, USA) injection (up to 2 mCi/kg IV, administered in a volume of <2 ml) into the saphenous vein. Subjects were returned to their cage for 30 min of conscious uptake. Pair mates were present in the cage during the baseline condition (see Table 1 for a summary of social condition in the home cage and during FDG uptake periods). During separation conditions, the subject was alone upon return to the cage. During the stranger encounter and partner reunion conditions, the subject was separated from the female by a mesh screen, which allowed viewing and limited tactile contact.

The same stimulus female was used for all stranger encounter conditions. This female had been previously hysterectomized and therefore was not ovulating while in the presence of subjects. She was observed closely during repeated exposures to stranger males; in all cases she did not display behavioral signals of stress. The nine stranger female scans occurred over 7 months. The minimum number of days between presentations of the female stranger to one of the subjects was 1 week, and the maximum was 72 days. Urine samples were collected from female pair mates and assayed for urinary estrone conjugates and pregnanediol-3α-glucoronide as previously described (Valeggia et al., 1999) to confirm *post hoc* that mates were not in estrous on the day of the reunion PET scan. Following the partner reunion or stranger scans, the subject male was in the same metabolism room as the stimulus female until the radiation decayed (less than 24 h), but without visual or tactile contact with the female. After the radiation decayed, both were returned to their respective cages in the colony room and the males were housed alone until their next scan. At the end of the series of PET studies, males were returned to social housing with their original pair-mate.

After the FDG uptake period, subjects were anesthetized with ketamine (25 mg/kg IM) and administered medetomidine (0.05 mg/kg IM). After the animal was sedated, a blood sample was collected from the femoral vein into a 3 ml heparin containing tube, and a sample of CSF was also collected. Both samples were immediately put on ice. An endotracheal tube was placed in the trachea, and a catheter was placed in the saphenous vein in order to administer IV fluids (lactated ringers solution, 10 ml/kg/h). Atipamazole was used to reverse medetomidine, and anesthesia was maintained with isoflurane (1–2%), while the male was positioned on the scanner bed feet first and the brain of the animal was positioned in the center of the scanner. PET imaging was performed on a microPET P4 scanner (Siemens Preclinical Solutions, Knoxville, TN, USA). Image acquisition began a mean of 66.80 (SD = 6.84) minutes post-FDG administration, and static PET scans were acquired for 60 min. Anesthesia was maintained throughout the scan. Animals were maintained in metabolism cages for 24 h after scanning, at which time radiation was decayed to background levels and animals were returned to their home cages/experimental conditions.

#### MRI Scan

Structural magnetic resonance imaging (MRI) scans were conducted in a GE Signa LX 9.1 scanner (General Electric Corporation, Milwaukee, WI, USA) with a 1.5 T field strength and a 3” surface coil. Each male was fasted 8–12 h before the procedure. At the start of the procedure, the male was sedated with ketamine (10 mg/kg IM) and medazolam (0.1 mg/kg IM), and an endotracheal tube was placed. A catheter was also placed in the saphenous vein in order to administer fluids as necessary. Images of the entire brain were collected. Anesthesia was maintained with isoflurane (1–2%) while the male was positioned in the MRI scanner. Each scan lasted approximately 20 min and consisted of a 3D SPGR pulse sequence in a coronal plane. Images of the entire brain were collected using the following parameters: echo time (TE) = 7.9 ms, repetition time (TR) = 22.0 ms, flip angle = 30.0º, field of view = 8 cm, number of excitations = 3, matrix = 256 × 256 and slice thickness = 1 mm. As a precautionary measure, the male’s EtCO2, oxygen saturation, heart rate and blood pressure were monitored throughout.

#### PET and MRI Coregistration and Quantification of FDG Uptake

**Figure 2:**
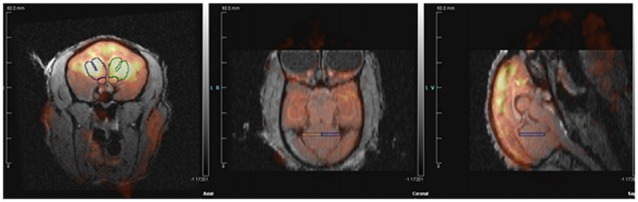
**Positron emission tomography (PET) image co-registered with structural magnetic resonance imaging (MRI)**.

Region of interest (ROI) structures were drawn on each subject’s MRI image using Siemen’s Inveon Research Workplace software (IRW, Siemens Healthcare, Malvern, PA, USA). Static PET images were reconstructed with a 3DRP reconstruction protocol. MRI images were co-registered with PET scan images using automatic rigid registration algorithm in IRW and checked visually for registration accuracy (Figure 2). Mean activity for the PET images were determined by applying the ROI drawn on the MRI images to the PET images in IRW. Data are presented in proportions of whole brain activity, calculated by dividing the mean activity in the ROI by mean activity of whole brain ROI. The effects on whole brain ROI were analyzed as mean activity (microcuries per cubic centimeter) rather than normalized units.

#### Blood Sampling and Hormone Analysis

Blood and CSF samples were collected immediately after anesthesia for the PET scan following the FDG uptake period, and placed on ice. Blood samples were collected at a mean of 5.13 min (SD = 2.96) after capture of the subject for the PET scan, and CSF samples were collected at a mean of 10.56 min (SD = 2.61) after capture. Blood samples in heparin containing tubes were centrifuged at 3000 RPM for 15 min at 4°C. Plasma was separated, and plasma and CSF samples were stored at −70°C until assay. CSF samples were assayed for AVP and OT. Plasma samples were assayed for AVP, OT, cortisol, insulin and glucose. There were no statistically significant relationships (*p* < 0.05) between the amount of time taken to collect the blood or CSF sample (“disturbance time”) and any of the hormone concentrations of the sample.

AVP and OT concentrations were estimated in duplicate using commercial enzyme immunoassay kits (Enzo Life Sciences, Farmingdale, NY, USA) previously validated for titi monkeys (Bales et al., 2005). Samples were not extracted prior to assay. Assay sensitivity was determined to be 2.34 pg/ml for AVP and 15.55 pg/ml for OT. Intra- and inter-assay coefficients of variation (CV) were 4.21% and 16.28% respectively for AVP, and 10.62% and 12.78%, respectively for OT.

Plasma cortisol concentrations were estimated in duplicate using commercial radioimmunoassay kits (Siemens Healthcare, Malvern, PA, USA). Prior to assay, samples were diluted 1:4 in PBS gel buffer. Assay procedures were modified with the addition of 0.5 and 2.35 μg/dl concentrations of standards along with the provided range of 1.0–49 μg/dl. Assay sensitivity has been determined to be 0.261 μg/dl. Intra- and inter-assay CV were 3.20% and 6.26%, respectively.

Plasma insulin was measured in duplicate using commercial ELISA kits (Ultra Sensitive Rat Insulin, Crystal Chem Inc., Downers Grove, IL, USA) as per manufacturer’s instructions for the wide range assay (0.1–12.8 ng/ml). This assay was validated for titi monkeys by assessing parallelism. The antibody used in this insulin assay had high cross-reactivity with other species as reported by the manufacturer. Intra- and inter-assay CVs were 5.17% and 12.86%, respectively. Plasma glucose was measured in duplicate using commercial glucose colorimetric assay kits (Caymen Chemical Company, Ann Arbor, MI, USA) as per manufacturer’s instructions. This assay was validated for titi monkeys by assessing parallelism. All samples were in detectable range of the assay. Intra- and inter-assay CVs were 2.28% and 3.70%, respectively.

### Data Analysis

#### Model Design

The predictor *condition*, based on experimental conditions, was used to model and compare categorical dummy-coded parameters, and the predictor *time*, based on measurement occasion, was used to account for sequential effects. These independent variables were modeled as:

1. Condition: baseline (reference) vs. four experimental conditions;
2. Time: measurement order, counterbalanced and unique to each subject.

These predictors were used in models of main and interacting effects to capture variance across two categories of continuous dependent variables. The first category of outcome variable was brain ROI, including: NAcc, VP, posterior cingulate cortex (PCC), LS, medial amygdala (MeA), medial preoptic area (MPOA), central amygdala (CeA), SON of the hypothalamus, PVN of hypothalamus, periaqueductal gray (PAG), cerebellum and whole brain. The second category of outcome variable was hormone concentration, including: plasma cortisol, plasma AVP, plasma OT, CSF AVP, CSF OT, plasma glucose and plasma insulin.

Two sets of analyses differentially assessed condition- and time-based effects, and fatherhood effects on our multiple physiological measures. The first set of analyses aimed to determine the hormonal and neural responses to separation from the pair mate. To examine the changes in physiological measures across conditions, we applied generalized estimating equations (GEE). Prior to conducting these analyses, we compared GEEs to linear mixed effects models (Muth et al., 2015) and confirmed that the GEEs were preferable due to problems with convergence of linear mixed effects models.

The second set of analyses examined the effects of fatherhood. These models did not contain order effects, but instead contained a variable indicating whether or not the male had fathered an offspring with his pair mate (*n* = 5 males that eventually had offspring for at least some scans) or not (*n* = 4) at the time of the scan. In this analysis, fatherhood models were dummy coded fatherhood as 0 = non-fathers, 1 = fathers. Therefore, the baseline reference was non-fathers.

#### Overview of GEE

GEE models are an appropriate choice for longitudinal psychological research, and can be applied to small-sample studies. These approaches are suited for repeated measures, due to their ability to account for dependency in outcome scores—an important feature of longitudinal data. Statistically, these data contain correlations, i.e., patterns of unique variation corresponding to each subject, which must not be ignored (Burton et al., 1998; Muth et al., 2015).

Unlike traditional *t*-tests, ANOVA, and simple regression models, which assume independence of residuals, GEEs account for the residual correlations across measurement occasions. These generalized models work with repeated measures to efficiently account for dependency in outcome measures without inflating sample size, distorting the true structure of the dataset (a consequence of ignoring residual correlation structure; Burton et al., 1998), or handling unbalanced designs with listwise deletion—a consequence of ordinary least squares methods such as the MANOVA approach to longitudinal data (Hedeker and Gibbons, 2006). GEEs are particularly relevant for small-sample, longitudinal studies with missing data. A key advantage of GEE models is to allow for inclusion of theory-driven correlation structures, to capture the dependency in repeated measures. In this study, our analyses use multiple GEE models with either one of two correlation structures (autoregressive or exchangeable), depending on whether the model variables included time. For models that accounted for time, we used an autoregressive correlation structure, which specifies diminishing correlation over time. For models that did not account for time, we used an exchangeable correlation structure, which specifies equal correlation across measures. This specification reflects a joint hypothesis: (a) observations within a subject are equally correlated across counterbalanced conditions (i.e., when ignoring measurement order); and (b) when accounting for order, correlations diminish at each subsequent measurement (i.e., scores from conditions that were measured further apart are less correlated than those measured closer together).

The subsequent models examined each condition in relation to baseline. Baseline condition served as our reference point to determine whether differences in stress-induced responses existed across long-term vs. short-term separation, and long-term vs. stranger or partner reunion conditions. Our focus is to study the unique effect of different partnership and separation conditions on the primates’ baseline measures of biological attachment and social stress markers. Moreover, individual conditions can be compared against each other (e.g., long-term separation vs. short-term separation) by comparing their differing magnitude of effects on baseline measures. Note that the various models presented here provide a multifaceted illustration of changes in baseline hormone concentrations and brain activity, however direct comparisons cannot be drawn across non-nested models (i.e., models with different predictors).

## Results

### Brain Regions of Interest: Responses to Separation and Reunion

**Figure 3:**
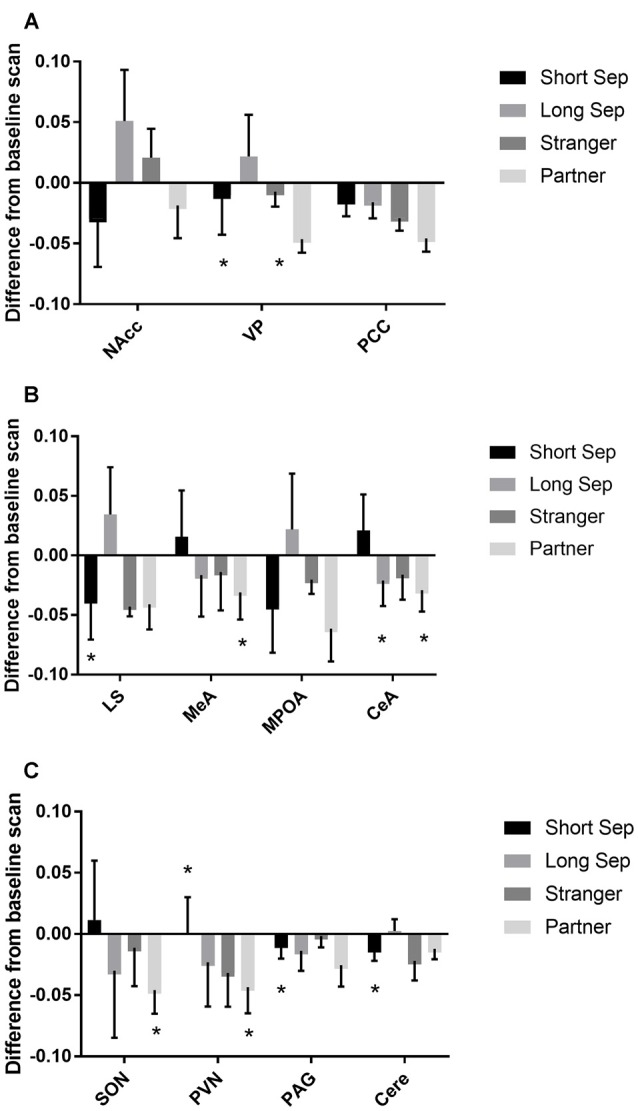
**Fluorodeoxyglucose (FDG) uptake by condition.** Please note that FDG in some areas also demonstrated a time-based effect, which is not shown on the graph (see Table 1). In some cases, this may be the reason that an effect designated as statistically significant may not appear so on the graph. **(A)** FDG uptake in the nucleus accumbens (NAcc), ventral pallidum (VP) and posterior cingulate cortex (PCC), in comparison to baseline. Conditions included a baseline scan, a short separation (short sep), a long separation (long sep), encounter with a strange female (stranger) and reunion with the partner (partner). *p < 0.05 for comparison to baseline concentrations. **(B)** FDG uptake in the lateral septum (LS), medial amygdala (MeA), medial preoptic area (MPOA) and central amygdala (CeA), by condition, in comparison to baseline. **(C)** FDG uptake in the supraoptic nucleus of the hypothalamus (SON), paraventricular nucleus of the hypothalamus (PVN), periaqueductal gray (PAG) and cerebellum (Cere); by condition, in comparison to baseline.

Among titi monkey males, FDG uptake in key neural regions of interest varied as a function of experimental condition and time (Table 2; Figure 3). In the short-term separation condition, as compared to baseline, FDG uptake was lower in the VP, LS, the PVN, the PAG and the cerebellum (Cere). The order of short-term separation vs. baseline, which were counterbalanced, also significantly predicted FDG uptake in the LS, MeA, MPOA, CeA, SON, PVN and Cere; with these areas showing higher FDG uptake when the short-term separation followed the baseline (Table 2; Figure 3).

**Table 2:** **Parameter estimates from generalized estimating equations (GEE) models—brain activity**.

	Generalized estimating equations: summary of significant predictors				
	Time Point*Condition				
	Value	Robust SE	df (df-resid)	Wald	Pr(>|W|)
**Nucleus accumbens**
Time*Short term sep	0.2842	0.1309	50 (46)	4.72	0.030
**Ventral Pallidum**
Time	−0.1577	0.0758	50 (48)	4.33	0.037
Short term sep	−1.0191	0.31	50 (48)	10.81	0.001
Stranger encounter	−1.7463	0.6102	50 (48)	8.19	0.004
Time*Short term sep	0.5676	0.1231	50 (48)	21.26	0.000004
Time*Stranger	0.4822	0.172	50 (48)	7.86	0.0051
**Posterior cingulate cortex**
Time	−0.4556	0.0895	50 (45)	25.91	3.60E^−07^
**Lateral septum**
Short term sep	−1.587	0.470	50 (40)	11.420	0.001
Time*Short term sep	0.723	0.143	50 (40)	25.690	0.000
**Medial amygdala**
Time	−0.302	0.145	50 (40)	4.350	0.037
Reunion pair mate	−2.891	0.566	50 (40)	26.110	0.000
Time*Short term sep	0.537	0.113	50 (40)	22.730	0.000
Time*Reunion pair mate	0.787	0.152	50 (40)	26.630	0.000
**Medial Preoptic Area**
Time*Short term sep	0.255	0.077	50 (40)	11.070	0.001
**Central amygdala**
Long term sep	−1.818	0.704	50 (40)	6.680	0.010
Reunion pair mate	−2.883	1.218	50 (40)	5.610	0.018
Time*Short term sep	0.643	0.122	50 (40)	27.750	0.000
Time*Stranger	0.559	0.218	50 (40)	6.550	0.011
Time*Reunion pair mate	0.748	0.240	50 (40)	9.670	0.002
**Supraoptic nucleus of the hypothalamus**
Reunion pair mate	−1.949	0.651	50 (40)	8.970	0.003
Time*Short term sep	0.508	0.127	50 (40)	16.050	0.000
Time*Reunion pair mate	0.503	0.190	50 (40)	7.050	0.008
**Paraventricular nucleus of hypothalamus**
Short term sep	−0.779	0.378	50 (40)	4.260	0.039
Reunion pair mate	−2.501	0.698	50 (40)	12.850	0.000
Time*Short term sep	0.395	0.130	50 (40)	9.240	0.002
Time*Reunion pair mate	0.636	0.184	50 (40)	11.970	0.001
**Periaqueductal gray**
Short term sep	−1.087	0.518	50 (40)	4.400	0.036
**Cerebellum**
Short term sep	−1.240	0.544	50 (40)	5.200	0.023
Time*Short term sep	0.517	0.266	50 (40)	3.770	0.052
**Whole brain**
Long term sep	−2.000	0.788	50 (40)	6.450	0.011

*Note. Only significant effects are reported*.

Long-term separation resulted in a significant reduction in glucose uptake in the CeA (Figure 3), as well as in whole brain FDG uptake. Exposure to a stranger female resulted in no significant main effects. However, there was an order effect on exposure to strangers in the CeA.

Presentation with the stranger female following the reunion with the pair mate increased FDG uptake in VP and CeA (Figure 3). Reunion with the pair mate resulted in a reduction in FDG uptake in the MeA, CeA, SON and PVN (Figure 3). When reunion with the pair mate followed the encounter with the stranger female, this significantly increased FDG uptake in the MeA, CeA, SON and PVN.

### Hormone Measures: Responses to Separation and Reunion

**Figure 4:**
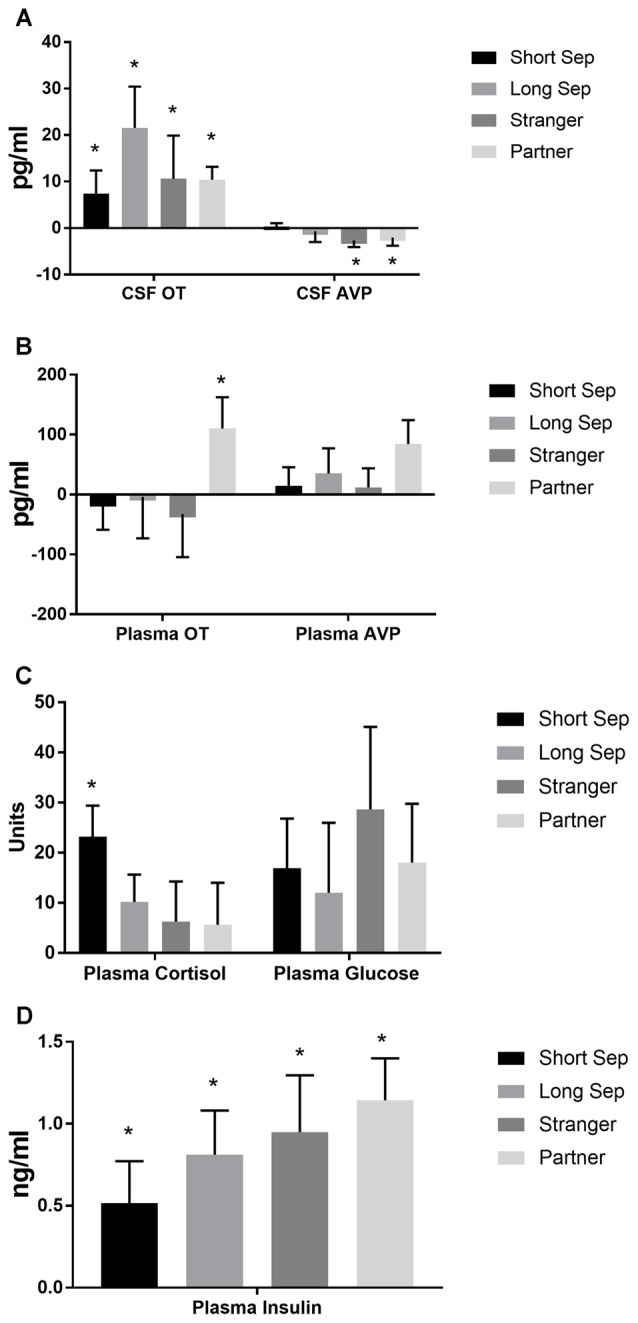
**Hormones by condition. (A)** Concentrations of oxytocin (OT) and vasopressin (AVP) in cerebrospinal fluid (CSF), in comparison to baseline. Conditions included a baseline scan, a short separation (short sep), a long separation (long sep), encounter with a strange female (stranger) and reunion with the partner (partner). *p < 0.05 for comparison to baseline concentrations. **(B)** Concentrations of plasma OT and AVP, in comparison to baseline. **(C)** Concentrations of plasma cortisol and glucose, in comparison to baseline. **(D)** Concentrations of plasma insulin, in comparison to baseline.

Significant parameter estimates for hormone outcome measures (based on scores standardized at the mean of baseline condition for each outcome measure) from GEE models emerged for time by condition (Table 3). In the short-term separation condition, as compared to baseline, plasma cortisol concentrations were higher (Figure 4), as were CSF OT and plasma insulin. In the long-term separation condition, plasma cortisol was no longer elevated, however, both CSF OT and plasma insulin concentrations remained elevated (Figure 4). Hormonal responses (not taking fatherhood into account, see below) were similar in reunion with the partner or exposure to a female stranger. CSF AVP was significantly lower in each of these conditions when compared to baseline, whereas CSF OT and plasma insulin were elevated compared to baseline (Figure 4).

**Table 3:** **Parameter estimates from GEE models—hormones**.

	Generalized estimating equations: summary of significant predictors				
	**Condition**				
	Value	Robust SE	df (df-resid)	Wald	Pr(>|W|)
**Plasma cortisol**
Short term sep	0.944	0.258	50 (48)	13.400	0.000
**CSF AVP**
Stranger	−1.132	0.409	39 (34)	7.680	0.006
Reunion pair mate	−1.096	0.474	39 (34)	5.341	0.021
**Plasma OT**
Reunion pair mate	0.872	0.342	49 (44)	6.500	0.011
**CSF OT**
Short term sep	0.772	0.348	42 (37)	4.933	0.026
Long term sep	1.276	0.507	42 (37)	6.336	0.012
Reunion pair mate	0.632	0.225	42 (37)	7.914	0.005
**Plasma insulin**
Short term sep	0.670	0.306	49 (44)	4.790	0.029
Long term sep	0.950	0.325	49 (44)	8.550	0.003
Stranger	1.220	0.431	49 (44)	8.000	0.005
Reunion pair mate	1.380	0.353	49 (44)	15.300	0.0000926

*Note. Only significant effects are reported*.

### Brain Regions of Interest: Effects of Fatherhood

**Figure 5:**
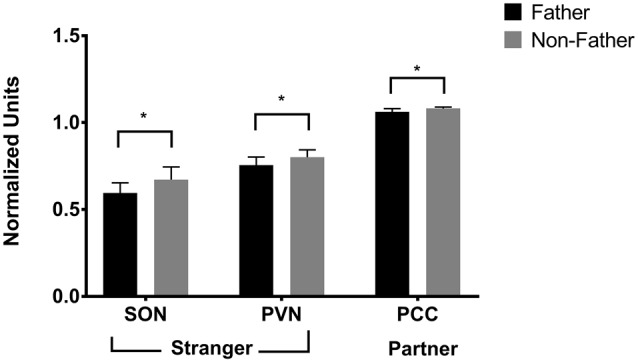
**FDG uptake by fatherhood.** FDG uptake in the SON (supraoptic nucleus of the hypothalamus) and the PVN (paraventricular nucleus of the hypothalamus) upon encountering a stranger female was higher in males that did not have offspring with their pair mate (non-fathers), than in males that did have offspring with their pair mate (fathers). *p < 0.05.

Fatherhood influenced FDG uptake during reunion with the partner compared to exposure to a stranger female (Table 4). When introduced to a female stranger, males that had successfully reproduced with their mate had lower FDG uptake in both the SON and the PVN, than males that had not successfully reproduced with their mate (Figure 5). When reunited with their pair mate, fathers had lower FDG uptake in the PCC than non-fathers (Figure 5).

**Table 4:** **Parameter estimates from GEE models (Brain)**.

	Generalized estimating equations: summary of significant predictors					
		Condition*Fatherhood				
Hormone	Source	Value	Robust SE	df (df-resid)	Wald	Pr(>|W|)
**Nucleus accumbens**
	Reunion pair mate (Non-father)	−0.3823	0.1503	30 (24)	6.47	0.011
**Ventral pallidum**
	Stranger	−0.5128	0.2596	30 (24)	3.9	0.048
**Posterior cingulate cortex**
	Stranger	−1.114	0.276	30 (24)	16.28	0.00006
	Fatherhood*Reunion pair mate	−1.649	0.539	30 (24)	9.35	0.002
**Lateral septum**
	Stranger	−1.0084	0.2709	30 (24)	13.86	0.0002
**Supraoptic nucleus of hypothalamus**
	Stranger	0.5562	0.1939	30 (24)	8.22	0.004
	Fatherhood*Stranger	−1.249	0.472	30 (24)	7.010	0.008
**Paraventricular nucleus of hypothalamus**
	Fatherhood*Stranger	−1.371	0.496	30 (24)	7.620	0.006
**Periaqueductal gray**
	Reunion pair mate (Non-father)	−0.7336	0.1719	30 (24)	18.22	0.00002
**Whole brain**
	Stranger	−0.82153	0.23044	30 (24)	12.71	0.00036

*Note. Only significant effects reported*.

### Hormone Measures: Effects of Fatherhood

**Figure 6:**
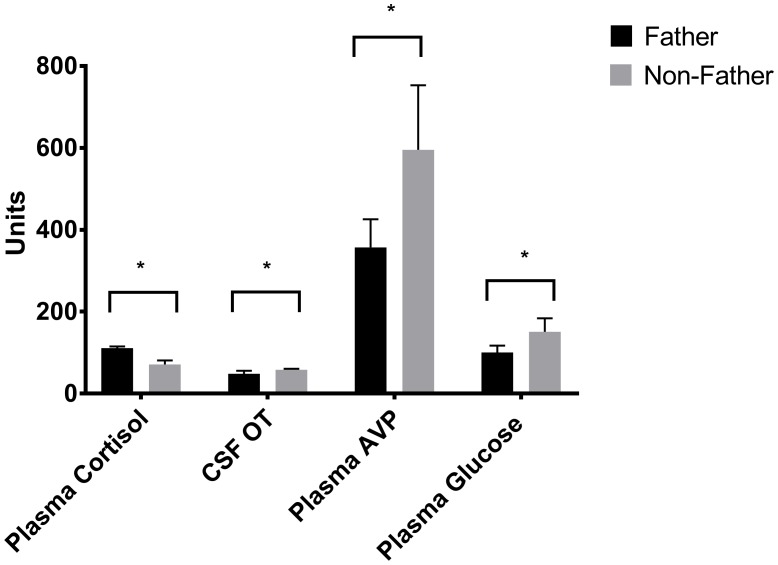
**Hormones by fatherhood.** Plasma AVP, CSF OT and plasma glucose were all significantly lower in fathers than non-fathers during a reunion with their pair mate; while plasma cortisol was higher. *p < 0.05.

Whether or not the pair had successfully had offspring predicted hormonal response to reunion with the partner (Table 5). In response to reunion with the female pair mate, males that had reproduced with her showed higher plasma cortisol concentrations, and lower CSF OT, plasma AVP and glucose concentrations, than non-fathers (Figure 6).

**Table 5:** **Parameter estimates from GEE models (Hormones)**.

	Generalized estimating equations: summary of significant predictors					
		Condition*Fatherhood				
Hormone	Source	Value	Robust SE	df (df-resid)	Wald	Pr (>|W|)
**Plasma Cortisol**
	Fatherhood*Reunion pair mate	1.305	0.510	30 (24)	6.560	0.010
**Plasma AVP**
	Reunion pair mate (non-father)	1.084	0.372	27 (21)	8.490	0.004
	Fatherhood*Reunion pair mate	−1.043	0.413	27 (21)	6.380	0.012
**CSF OT**	
	Reunion pair mate (non-father)	0.830	0.278	24 (18)	8.910	0.003
**Plasma Glucose**
	Reunion pair mate (non-father)	1.676	0.758	30 (24)	4.890	0.027
	Stranger	2.404	1.169	30 (24)	4.230	0.040
	Fatherhood*Reunion pair mate	−1.655	0.812	30 (24)	4.150	0.042
**Plasma Insulin**
	Reunion pair mate (non-father)	1.439	0.656	29 (23)	4.810	0.028
	Fatherhood (baseline)	−1.091	0.397	29 (23)	7.540	0.006

*Note. Only significant effects reported*.

## Discussion

**Figure 7:**
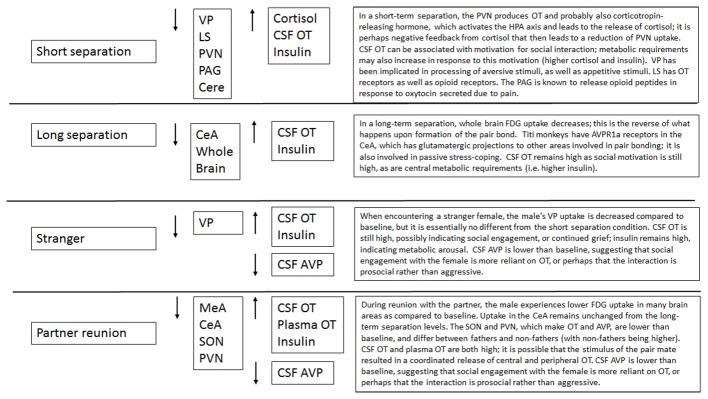
**Summary of effects of each condition**.

Male titi monkeys showed widespread effects of separation and reunion on brain FDG uptake and central and peripheral hormones. In many cases, the neural changes appear to be potentially attributable to the reactions of the OT and AVP systems, or to opioids acting on κ opioid receptors. Observing the patterns of change in FDG uptake and hormone concentrations aids our understanding of these challenges to the pair bond (summarized in Figure 7). Notably, in many of our models, the order in which we performed our PET scans was statistically significant. This argues for continued attention to careful counter-balancing of conditions whenever possible, as well as inclusion of order effects in statistical models.

**Figure 8:**
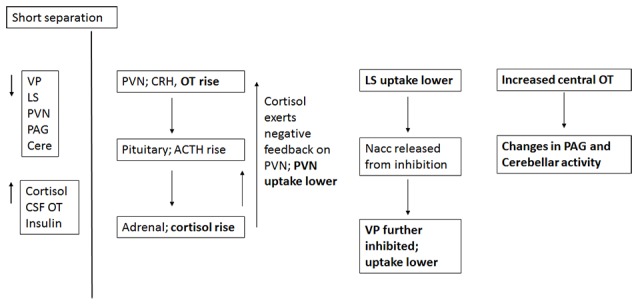
**Proposed relationships of neural and hormonal changes associated with short separation.** The parts that are in bold are data from this study; the other relationships are hypothesized.

Short-term separation from the pair mate resulted in a multitude of neural and hormonal changes (Figure 8). FDG uptake was lower than baseline in many regions of interest, particularly those in which OT and AVP are synthesized (PVN) or which have OT or AVP receptors (LS, PAG, Cerebellum) in the titi monkey brain (Ragen and Bales, 2013; Freeman et al., 2014). This is somewhat counter-intuitive, as we might expect *increases* in some of these areas. However, reductions in PVN uptake during short separations may have been associated with the production of CRH, activation of the hypothalamo-pituitary-adrenal axis and the subsequent release of cortisol, which was elevated during short separations. Cortisol may have then exerted negative feedback on CRH production, leading to the depression in glucose uptake in the PVN. Plasma cortisol and insulin, which were also elevated, can be associated with both metabolic arousal/readiness and psychological stress (Gold, 2015; Mendoza, 2017).

The LS also showed a decrease in FDG uptake during short-term separation. The LS in titi monkeys is a nexus for many neurotransmitter systems; it has receptors for OT (Freeman et al., 2014), both μ and κ opioid receptors (Ragen et al., 2015a), and most likely dopamine receptors as well (Sheehan et al., 2004). It also exerts an inhibitory influence on the NAcc via GABAergic afferents (Sheehan et al., 2004); whereas the NAcc exerts an inhibitory influence on the VP (Numan and Young, 2016). One possibility is that decreased FDG uptake in the LS resulted in lowered inhibition in the NAcc, which then resulted in increased inhibition in the VP (and lower FDG uptake there). One problem with this hypothesis is that in this study, NAcc FDG uptake did not change during short separations; it is possible that our timeline was not optimal for detecting this change.

As stated above, there were no changes in FDG uptake in the dopaminergic NAcc, which has AVPR1a receptors in titi monkeys, and which has been implicated in human and vole studies of grief (O’Connor et al., 2008; Bosch et al., 2016). Short-term separation, however, did result in a reduction in FDG uptake in the closely related, also dopamine-innervated VP, which does not have either OXTR or AVPR1a in titis (Freeman et al., 2014). Neither do titi monkeys have μ opioid receptors in VP; however, they do have κ opioid receptors (Ragen et al., 2015a). κ opioid receptors are associated with negative affect and the dysphoria of social separation in vole and titi monkeys (Resendez et al., 2012; Resendez and Aragona, 2013; Ragen et al., 2015b). κ opioid receptors are also present in titi monkey LS (Ragen et al., 2015a), although they have not been examined for titi monkey midbrain or cerebellum. These commonalities suggest a role for dopamine and κ opioid receptors in the response to short-term separation between pair mates in titi monkeys. Future studies should include measurement of opioid peptides (such as dynorphin), in CSF.

Finally, CSF OT concentrations were also elevated with short-term separation. Prairie voles that undergo a social challenge (isolation) have also shown central elevations in OT (Grippo et al., 2007; Sun et al., 2014), and humans that are in a distressed pair relationship showed increased plasma OT (Taylor et al., 2010). Taken together with the results of neural glucose uptake, we infer that male titi monkeys undergoing an acute separation from their pair mate are releasing OT, which then is binding to OTR in the LS, and AVPR1a in the PAG and cerebellum, since OT and AVP can bind to each other’s receptors (Barberis and Tribollet, 1996). OT in the PAG is known to lead to the release of opioids in response to pain (Ge et al., 2002; Yang et al., 2011); perhaps the response is similar for “social pain”. This release of OT may be a coping strategy to deal with the pain of separation while increasing the motivation to interact socially to locate the mate, or perhaps find a new mate.

Long-term separation resulted in fewer changes than short-term separations, in FDG uptake or hormone concentrations, when compared to baselines. CSF OT, as well as plasma insulin, remained elevated; again probably associated with motivation to travel and engage in social interactions. In addition, whole brain FDG uptake was lower. This represents the reverse of a shift that occurs in whole brain FDG uptake at the formation of the pair bond, when they increase significantly and remain high (Bales et al., 2007; Maninger et al., unpublished data). We have found that this increase occurs between 2–7 days post-pairing (Maninger et al., unpublished data). The decrease in whole brain FDG uptake in this study occurs along a similar timeline, 2–14 days post-pairing, indicating a similar timeline for whole brain metabolism to adjust to a drastic change in social circumstance. Finally, with long separations we see a reduction in glucose uptake in the CeA, which has dense AVPR1a binding in titi monkeys and has been associated with passive stress-coping in other species (Ebner et al., 2005; Freeman et al., 2014).

In our time*condition models, reunion with the pair mate involved a decrease in glucose uptake in the MeA, CeA, SON and PVN when compared to baseline. The MeA typically inhibits other areas involved in social behavior, so a reduction in uptake there may indicate that these other areas could escape inhibition. The CeA did not really change when compared to the long-term separation level, so this is probably not a specific reaction to the pair-mate. Changes in SON and PVN were mediated by fatherhood (see below). Males also experienced a reduction in plasma AVP, an increase in CSF and plasma OT and an increase in insulin. Reunion with the pair mate was our only condition to show a coordinated increase in both CSF and plasma OT. Plasma OT is probably most synchronized with CSF OT in the case of a potent social stimulus (Landgraf and Neumann, 2004; Feldman et al., 2011; Kenkel et al., 2012). Reductions in plasma AVP were mostly present in fathers (Figure 6).

Fatherhood mediated the neural and hormonal effects observed following reunion with the pair mate and exposure to a female stranger. Upon reunion with the pair mate, fathers experienced a reduction in FDG uptake in the PCC when compared to non-fathers. Fathers also displayed higher cortisol but lower plasma AVP and plasma glucose, than did non-fathers. In a recent study in which human males chose a dance partner vs. a non-social choice situation, the PCC was specifically activated by partner choice (Yokoyama et al., 2016). This may suggest that non-fathers are more open to a new partner than fathers. Prairie vole males whose mates do not become pregnant in a short time-frame show less aggression towards novel females (Resendez et al., 2016).

The possibility that pair bonds are weaker in males that have not yet reproduced with their mates was also supported by neural differences during the stranger encounter; when encountering a stranger female, fathers displayed lower FDG uptake in the SON and PVN. Non-fathers that have been separated from their mate for an extended period are possibly initiating the formation of a new pair bond with the stranger female. In other words, since non-fathers may not be as bonded to their absent pair mate, when they are exposed to a stranger female there is greater activity in the SON and PVN, which might result in a release of neuropeptides to encourage the formation of a bond with the new female. While we have studied the neurobiology and physiology of pairing in male titi monkeys (Bales et al., 2007), we have not yet studied the changes that occur within minutes or hours of pair formation or the initiation of parenting—which may prove to be a particularly sensitive period for neurobiological cascades and subsequent regulation. The observed mediating effect of fatherhood on the neuroenergetics and neurobiological cascades within this primate taxon contributes to the growing body of literature around the biology of fatherhood (Fernandez-Duque et al., 2009; Bales et al., 2011; Gettler, 2014; Saltzman and Ziegler, 2014; Bales and Saltzman, 2016).

Overall, our results suggest that mechanisms for maintenance of the pair bond in socially monogamous titi monkeys may rely on similar systems as they do in prairie voles. Regions of the mesolimbocortical system, such as the VP, areas of the extended amygdala that facilitate social memory, such as the LS and areas that produce OT and AVP (PVN and SON) seem to be of particular importance for the maintenance of pair bonds in both the rodent and primate species; avoidance of separation being one behavior that leads to sustained bonding. The specifics, however, probably differ according to species, as do distributions of the receptors for OT and AVP (Freeman and Young, 2016). The regional similarities but mechanistic differences are expected in the context of convergent evolution by which selection acts on similar structures and processes in ways unique within particular lineages. Studies in humans should take into account emerging information regarding the distribution of these receptors in humans (Freeman et al., 2016b). Future studies should also use neurochemically specific PET tracers, where available, to more closely identify the specific peptide and monoamine systems involved in various aspects of attachment. Finally, the multitude of neural and hormonal changes associated with separation and reunion with the pair mate argue for the salience and impact of these manipulations as social stressors.

## Author Contributions

The research was designed by KLB, SPM, WAM, KH and NM. Research was carried out by KH, NM, BJR, RHL, MRJ, SPM, MLF-P and KLB. SRC was responsible for imaging data. CM and EF performed data analysis. KLB, NM and KH wrote the manuscript, which was edited by all authors.

## Conflict of Interest Statement

The authors declare that the research was conducted in the absence of any commercial or financial relationships that could be construed as a potential conflict of interest.
